# Characterization of yellow root cassava and food products: investigation of cyanide and β-carotene concentrations

**DOI:** 10.1186/s13104-020-05175-2

**Published:** 2020-07-11

**Authors:** Chiemela S. Odoemelam, Benita Percival, Zeeshan Ahmad, Ming-Wei Chang, Dawn Scholey, Emily Burton, Polycarp N. Okafor, Philippe B. Wilson

**Affiliations:** 1 School of Animal, Rural and Environmental Sciences, Nottingham Trent University, Brackenhurst Campus, Nottingham, NG25 0QF UK; 2 Faculty of Health and Life Sciences, De Montfort University, The Gateway, Leicester, LE1 9BH UK; 3 Nanotechnology and Integrated Bioengineering Centre, University of Ulster, Jordanstown Campus, Newtownabbey, BT37 0QB Northern Ireland, UK; 4 Department of Biochemistry, Michael Okpara University of Agriculture, Umudike, Nigeria

**Keywords:** Cyanide, β-carotene, Cassava, Nutrition, Flours

## Abstract

**Objective:**

Cyanide is a highly toxic compound, and the consumption of products containing cyanide is a significant public health concern. Conversely, *β*-carotene possesses essential nutritional attributes for human health, therefore the characterisation and quantification of both compounds in food products is fundamental. Herein, cyanide and β-carotene levels in two flours produced from the roots of two varieties of cassava (*Manihot esculenta crantz*), namely UMUCASS-38(TMS 01/1371) and NR-8082, and their associated food products were detected and quantified.

**Results:**

The cyanide content of NR-8082 and UMUCASS-38 flours was determined at 18.01 ± 0.01 ppm and 17.02 ± 0.02 ppm (mean ± SD), respectively. These flours contained significantly higher (*p *< 0.05) than the residual cyanide levels determined in the cookies and cake produced therefrom with levels of 10.00 ± 0.00 ppm and 7.10 ± 0.14 ppm (mean ± SD), respectively. The levels of β-carotene determined in both the cake and cookie samples varied significantly (*p *< 0.05). The highest levels of β-carotene at 6.53 ± 0.02 µg/g (mean ± SD) were determined in raw roots of UMUCASS-38. While NR-8082 levels of β-carotene were less than UMUCASS-38 at 1.12 ± 0.02 µg/g (mean ± SD). Processing the roots into flour reduced the β-carotene content to 4.78 ± 0.01 µg/g and 0.76 ± 0.02 µg/g (mean ± SD) in UMUCASS-38 and NR-8082 flours, respectively. Cookies and cake produced from flour derived from the UMUCASS-38 variety had (mean ± SD) 2.15 ± 0.01 µg/g and 2.84 ± 0.04 µg/g of β-carotene, respectively.

## Introduction

**Fig. 1:**
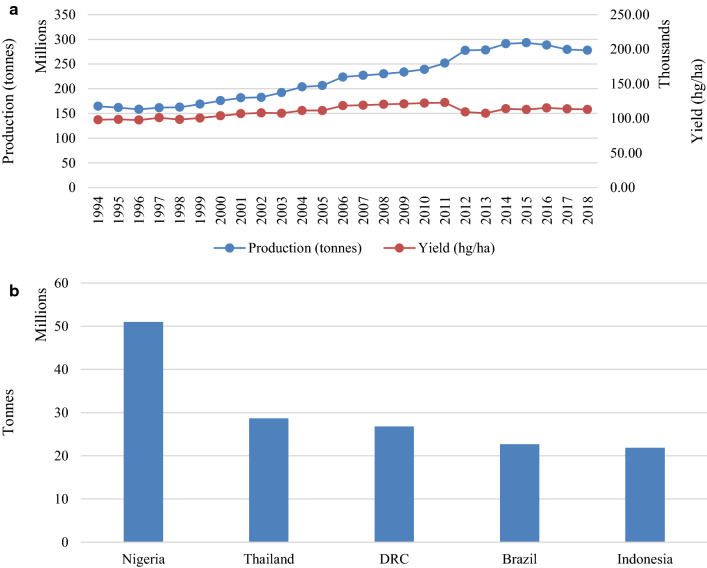
World cassava production statistics. **a** Production/yield quantities of cassava in the world from 1994 to 2018 in tonnes. **b** Top 10 producers of cassava from 2008 to 2018 with data presented in tonnes. Data source: FAOSTAT [1]

There has been a substantial increase in world production of cassava since 2001, with production reaching a peak of 293.01 million tonnes in 2015 (Fig. 1a) [1]. According to FAOSTAT [1], world cassava production for the year 2018 is estimated to be approximately 277.81 million tonnes. The top five countries for cassava production were Nigeria, Thailand, Democratic Republic of Congo (DRC), Brazil and Indonesia (Fig. 1b) [1].

Cassava has been reported to contain 25 mg vitamin C, 40 mg phosphorus and 50 mg of calcium per 100 g of plant material [2]. The concentration of proteins, riboflavin, thiamine and niacin in cassava is very low in comparison to other tuber crops, thus making cassava one of the highest sources of carbohydrates among tuber crops [3]. The carbohydrate content of cassava ranges from 64 to 72% starch (amylose and amylopectin), the starch present in cassava is structurally different from that found in cereal; in its branch chain length distribution, amylose content and its granular structure. Approximately 17% of sucrose is also found in cassava, predominantly in the sweet varieties, and limited quantities of fructose and dextrose have also been reported. The protein content is between 1 and 2%, with low essential amino acid profiles, particularly methionine, tryptophan and lysine. Furthermore, cassava possesses a high dietary fibre content (3.40–3.78% soluble, and 4.92–5.6% insoluble) [4, 5].

### Cyanogenic glycosides and cyanide

**Scheme 1:**
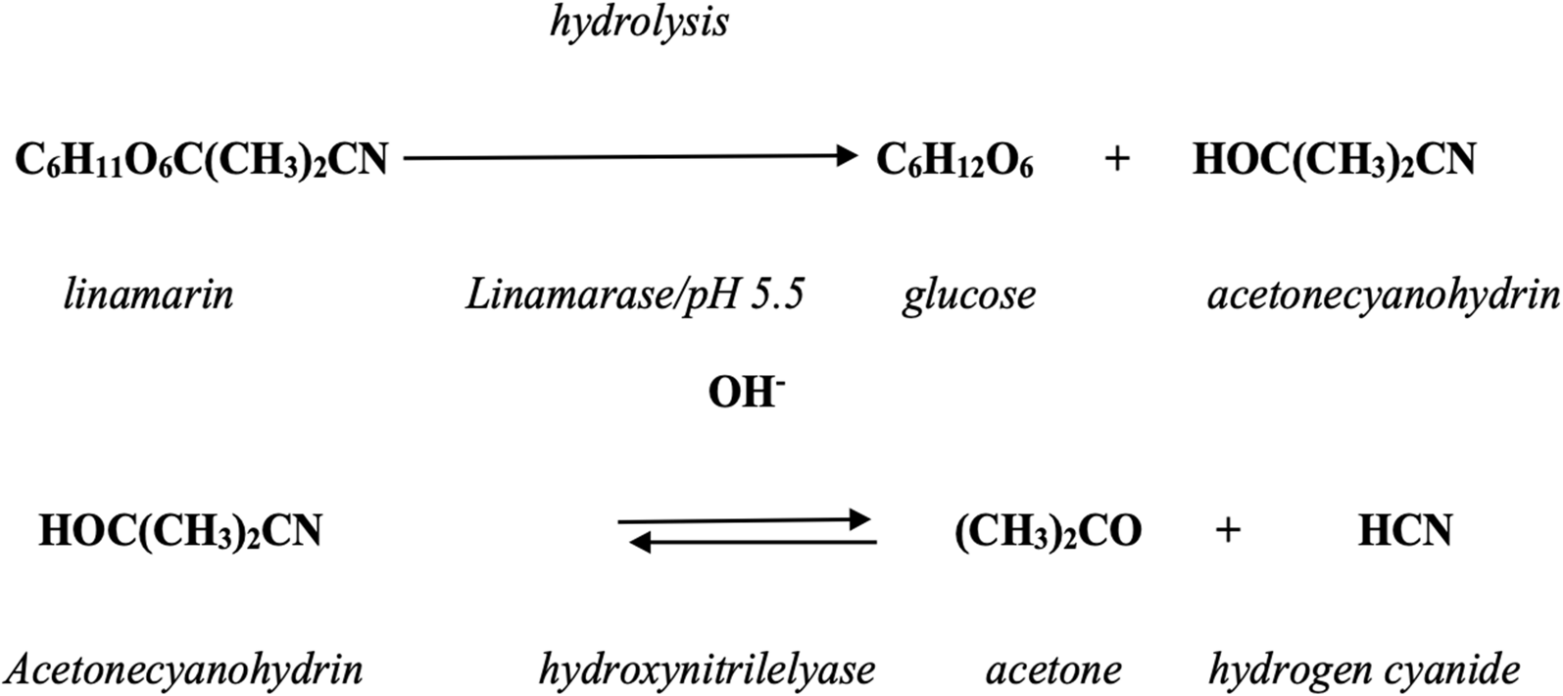
Hydrolysis of linamarin adopted from Idibie, 2006 [12]

Cyanogenic glycosides are present in all parts of the plant with the leaves having the highest concentration [6]. According to Kotopka and Smolke [7], these compounds act as chemical defences produced by the plants. Cyanogenic glycosides act as a deterrent against pathogenic organisms and the activities of herbivores. Cassava is composed of two cyanogenic glycosides namely lotaustralin and linamarin which release hydrogen cyanide (HCN) upon destruction of the tissues. Destruction of tissues occurs as a result of mechanical damage during harvesting, or indeed chewing action of herbivores and other consumers. The presence of these glycosides, especially in the tuber, has been attributed to the extreme conditions in which the crop is grown, with drought being one of the parameters investigated thus far. Findings from research monitoring cassava toxicity in Mozambique, showed that the levels of residual cyanide tripled during drought years in comparison to the normal years [8, 9]. The breakdown of linamarin catalysed by an endogenous β-glucosidase (linamarase) due to the disruption of cellular integrity of a plant cell leads to the formation of a cyanohydrin and a sugar (Scheme 1). The cyanohydrin formed, is highly unstable under neutral conditions and undergoes further decomposition to yield an aldehyde, or a ketone and cyanide [10–12]. The enzyme hydroxynitrile lyase catalyses the breakdown of the cyanohydrin forming a carbonyl compound and hydrogen cyanide (Scheme 1) [12, 13]. Cyanogenic glycoside is toxic upon degradation as it is catalysed by its endogenous β-glucosidase to yield hydrogen cyanide, the consumption of poorly processed foods which contain cyanogenic glycosides would eventually lead to acute cyanide poisoning (LD_50_ of 1.52 mg/kg for oral administration) [14].

High levels of cyanide intake associated with the chronic consumption of cyanogenic glycosides (from cassava etc.) are reported to lead to diseases such as iodine deficiency disorder, tropical ataxic neuropathy and konzo [15, 16].

Cassava being of a lower nutritional value than other staple foods consumed in sub-Saharan Africa and vitamin A deficiency being a major hindrance to improved nutrition [16]. This prompted the biofortification of cassava, giving rise to the genetically engineered pro-vitamin A cassava developed under the IITA-Harvest Plus program [16, 17]. This was rationalised to partially address the vitamin A deficiency affecting much of the sub-Saharan Africa population [16]. There is approximately 23,500 child mortalities annually in Kenya as a result of micronutrient deficiencies as school children often suffer from sub-clinical vitamin A deficiency [16, 18]. Herein, we determine the levels of residual hydrogen cyanide and β-carotene content as yellow flesh cassava UMUCAS 38 (TMS 01/1371) is being processed from tuber into confectionary products whilst NR-8082 is used as control sample.

## Materials and methods

### Materials

Acetone, hyflosupercel (Celite), 3 mm Whatman filter paper, vacuum filtration equipment, UV visible spectrophotometer (Jenway 6300, Staffordshire, UK). All chemicals within this study were purchased from Sigma Aldrich (1 Friesland Drive Longmeadow Business Estate 1645 Modderfontein South Africa).

### Sample preparation

Freshly harvested roots of the two experimental cultivars UMUCASS-38 (TMS 01/1371) and NR-8082 (Control) were obtained from the Cassava Programme of National Root Crops Research Institute (NRCRI), Umudike, Nigeria. The samples were processed into high quality cassava flour (HQCF) following the methods described by Onabolu et al. [19] and oven dried at a temperature of 115 °C for 6 h. The HQCF sample was further processed into consumer products.

### Carotenoid determination

The extraction with acetone for carotenoid analysis developed by Rodriguez-Amaya and Kimura [20] was used for the determination of the total β-carotene content of the samples. 5 mg of the sample was ground with the aid of hyflosupercel (3.0 g) in 50 ml of cold acetone and vacuum filtered. The filtrate was extracted using 40 ml petroleum ether (PE). Saturated sodium chloride was used to prevent the formation of emulsion. The lower aqueous phase was discarded while the upper phase was collected and filtered through 15 g of anhydrous sodium sulphate to eliminate residual water. The separating funnel was washed with PE and the flask was made up to 50 ml. The absorbance of the solution was measured at 450 nm and the total carotenoid content was calculated using the Beer-Lambert law (Eq. 1).

1 $$ Total \;carotenoid \;content \;\left( {ug/g} \right) = \frac{{A \; \times \;Volume \;\left( {\text{ml}} \right)\; \times 10^{4} }}{{A\frac{1\% }{{1\;{\text{cm}}}} \; \times \,sample weight \;\left( {\text{g}} \right)}} $$

where A = absorbance; volume = total volume of extract (50 or 25 mL); $A\frac{1\% }{{1\;{\text{cm}}}}$ = absorption coefficient of β − carotene in PE (2592).

### Cyanide determination

The simple picrate paper method was used to determine the levels of residual hydrogen cyanide [21]. The picrate paper method was preferred to high-end instruments due to being inexpensive, and requiring only small amounts of sample. Moreover, portability of analytical instruments is a growing trend in routine food analytical techniques and this approach could be performed with a portable UV analytical instrument [22].

However, there are advantages to using high-end instruments, such as potentiometry/amperometry/ion chromatography-pulsed amperometry, for quantification and detection which include increased accuracy and sensitivity [23]. 100 mg of the sample was placed in a flat-bottomed plastic bottle containing the enzyme (linamarinase), buffer and picrate paper. The contents were left to incubate in the dark for 24 h at room temperature. The picrate papers darkened as a result of cyanide production were then placed in a test tube with 5 ml of distilled water. The sample was allowed to stand at room temperature for 30 min. The UV absorbance was determined at a wavelength of 510 nm and total cyanide content calculated according to Eq. 2.

2 $$ Total \;cyanide\; content \;\left( {\text{ppm}} \right) = 396 \; \times \;Absorbance $$

### Statistical analysis

Paired t-tests were carried out to compare the levels of cyanide and β-carotene in the different samples using Prism 8 (Graph Pad software LLC). ANOVA was carried out using the Statistical Package for Social Sciences (SPSS), version 22. Statistical significance was set at *p *< 0.05.

## Results

### Cyanide determination

**Fig. 2:**
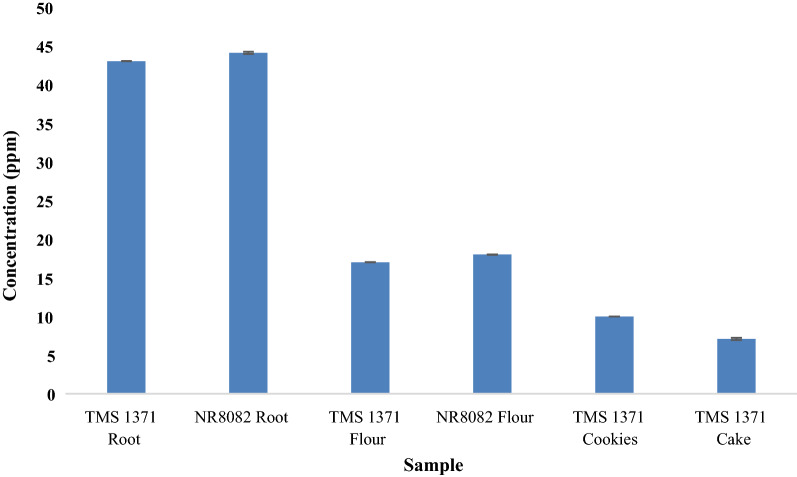
Levels of residual cyanide (with standard deviation error bars) in roots and products determined using simple picrate paper method

Fresh NR-8082 carried the highest cyanide concentration of 44.10 ± 0.14 ppm (mean ± SD) while the fresh UMUCASS-38 had a value of 43.02 ± 0.02 ppm (mean ± SD) (Fig. 2). NR-8082 flour was determined as having the highest cyanide level of 18.01 ± 0.01 ppm (mean ± SD) while the UMUCASS-38 had the least at 17.02 ± 0.02 ppm (mean ± SD). The cookie sample showed the highest concentration at 10.00 ± 0.00 ppm (mean ± SD) as compared to the cake sample with 7.10 ± 0.14 ppm (mean ± SD). In addition, the NR-8082 variety had significantly higher cyanide concentration (*p *< 0.05) than the yellow flesh (Fig. 2).

### Carotenoid determination

**Fig. 3:**
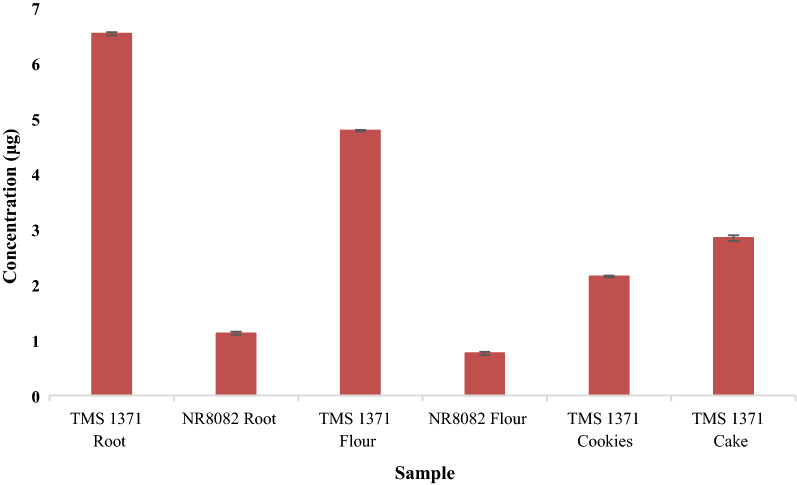
Levels of *β*-carotene (with standard deviation error bars) in roots and products determined using the extraction with acetone method for carotenoid analysis developed by Rodriguez-Amaya and Kimura

Fresh UMUCASS-38 possessed a carotenoid content of 6.53 ± 0.02 µg/g (mean ± SD) compared to that of the NR-8082 variety at 1.17 ± 0.02 µg/g (mean ± SD). The products retained a portion of the β-carotenoids after production; the cake sample had a residual β-carotene concentration of 2.84 ± 0.04 µg/g whilst that of the cookie sample was determined at 2.15 ± 0.01 µg/g (mean ± SD) (*p *< 0.05) (Fig. 3).

## Discussion

Chronic exposure to cyanide causes a myriad of cardiac, neurological and metabolic dysfunctions which can be fatal [24]. As a result of concern regarding the levels of potential residual cyanide remaining in cassava after processing, the roots were classified according to their potential toxicity to humans and animals as non-toxic (less than 50 mg HCN kg^−1^ in fresh root), moderately toxic (50–100 mg HCN kg^−1^ in fresh root) and highly toxic (above 100 mg HCN kg^−1^ in fresh root) [25]. The lethal dose of cyanide in humans is in the range of 0.5 to 3.5 mg/kg body weight [9, 26]. The level of cyanide in the flour within this study was reduced by almost 60% as a result of food processing. The food products, cakes and cookies, possessed lower levels of cyanide. This is acceptable according to the World Health Organisation (WHO) standard of 10 ppm [11]. This WHO standard of 10 ppm or 10 mg HCN/kg body weight was reached as a result of the lack of quantitative and epidemiological information to estimate a safe level. However, the Joint FAO/WHO Expert Committee on Food Additives (JECFA) concluded that up to a level of 10 mg HCN/kg body weight (10 ppm) in the codex standard of cassava flour is not associated with acute toxicity [27]. The low cyanide levels in the products was as a result of the processing method which involved the peeling, grating and subsequent oven drying to produce HQCF. The low cyanide levels in the products suggest that the food products may not be highly toxic to consumers when employing the WHO standard as a benchmark [11]. The body has several pathways for the detoxification of cyanide, and this primarily involves the conversion of soluble thiocyanate (SCN^−^) by the enzyme rhodanese [27]. Lesser pathways of metabolism include the complexation of cyanide with cobalt in hydroxocobalamin to form cyanocobalamin (Vitamin B12) [27].

The consumption of these cassava varieties as a staple food must be complemented by a diet rich in protein from exogenous sources due to the low protein content of cassava itself. The findings of the current study showed a reduction in cyanide and β-carotene levels in the processed products (Figs. 2 and 3). The levels of β-carotene after processing using a method which has been confirmed to reduce cyanide levels at the expense of leaching or destruction of essential nutrients such as vitamin C, β-carotene (vitamin A precursor) and vitamins B (riboflavin, niacin and thiamine) suggests that the consumption of yellow root cassava UMUCAS 38 does indeed contribute to the recommended daily allowance of vitamin A [9]. The continuous consumption of cassava-based products without sufficient protein intake limit protein synthesis, thus being related to stunted growth in children whose diets are based on this as a major nutrition source [25].

Carotenoids, a class of colourful plant pigments that the body can convert to vitamin A, are also powerful antioxidants that have been suggested to contribute to the resistance against certain forms of cancer and heart diseases, and also enhance immune response to infections [25]. The predominant carotenoid in yellow cassava being β-carotene, suggests a need for dietary supplementation as the consumption of this yellow root cassava may not meet the recommended daily allowance (RDA) for vitamin A in men (750–900 µg daily), women (700 µg daily) and children (400–600 µg daily) [25, 28].

The carotenoid content of fresh UMUCASS-38 and the NR-8082 variety were 6.53 µg/g and 1.12 µg/g, respectively. There was a decrease in the carotenoid content in the flour level as a result of exposure to light and heat treatment. There was also a significant decrease in the HCN levels which can be attributed to further heat treatment i.e. baking and mixing which could lead to the release of the enzyme linamarinase.

In conclusion, food products contained less residual cyanide and β-carotene than their fresh counterparts. The study demonstrates the viable food safety of cassava-based products for human consumption, as well as the need to supplement vitamin A from exogenous sources. Cassava products provide potential promise as a source of vitamin A in developing countries, which is important for deficient regions, where cassava could be used as a staple food. Based on the findings from this investigation, further agronomic studies should be carried out in order to continuously improve the β-carotene content of these biofortified cassava varieties, whilst ensuring a low biogenic content.

## Limitations

- Using the simple picrate paper method for cyanide determination is limited by the rate of reaction of approximately 16–24 h for completion. The chemicals require special handling and storage, the results obtained can sometimes be indefinite. Hence, the dissolving of the chromophore from the picrate paper for a quantitative determination using a spectrophotometer.

## Data Availability

All data generated or analysed during this study are included in this published article.

## Abbreviations

FAO: Food and Agriculture Organization
IITA: International Institute of Tropical Agriculture
HQCF: High quality cassava flour
ANOVA: Analysis of variance
SPSS: Statistical Package for Social Sciences
DRC: Democratic Republic of Congo
P.E.: Petroleum ether
JECFA: Joint FAO/WHO Expert Committee on Food Additives
